# New perspectives and evolution of ovulation synchronization protocols in bovine females

**DOI:** 10.1590/1984-3143-AR2025-0048

**Published:** 2025-08-14

**Authors:** José Nélio de Sousa Sales, Laís Reis Carvalho, Luiz Manoel Sousa Simões, Lucas Araujo Lemos, Matheus Pedroso Vicente, Rafael Resende Rabelo Silva, Luísa Oliveira Orlandi, Pietro Sampaio Baruselli, José Camisão de Souza

**Affiliations:** 1 Universidade Federal de Juiz de Fora – UFJF, Departamento de Medicina Veterinária, Juiz de Fora, MG, Brasil; 2 Universidade Federal de Lavras – UFLA, Faculdade de Zootecnia e Medicina Veterinária, Lavras, MG, Brasil; 3 Universidade de São Paulo – USP, Faculdade de Zootecnia e Medicina Veterinária, São Paulo, SP, Brasil

**Keywords:** ovulation, estrus, synchronization, reproduction, cow

## Abstract

The productivity in livestock systems is related to the reproductive efficiency of herds. Over the years, strategies have been developed to improve the reproductive rates of female cattle. Initially, estrus synchronization protocols were developed, however, difficulties related to prolonged postpartum anestrus and estrus observation resulted in low conception rates in these programs. Subsequently, hormonal associations were used to synchronize ovulation and inseminate female cattle at a predetermined time, eliminating the need for estrus observation and improving the fertility rates of cows in postpartum anestrus. Several adjustments were made to improve the response to a timed-artificial insemination (TAI) protocol in different production systems and animal categories. Finally, the development of recombinant drugs and nanotechnology may optimize production systems. Thus, the objective of this review is to detail the research carried out over the years related to the evolution of TAI protocols.

## Introduction

Reproductive assisted technologies stand out on the global stage for providing increased genetic gain and higher productivity in cattle herds. In livestock systems, these characteristics are directly related to the reproductive efficiency of bovine females (Baruselli et al., 2025b). Among these biotechniques, timed-artificial insemination (TAI) is well-established and widely used with the aim of intensifying reproductive management. In Brazil, the TAI market resumed growth in 2024 after two years of decline, accounting for 91.8% of the inseminations performed. Additionally, there was an increase of 2.6% (25,346,470 doses) and 3.3% (23,267,777 protocols) in the commercialization of semen doses and TAI protocols compared to previous years (Baruselli, 2025a).

Initially, TAI protocols were based on synchronizing the emergence of a new follicular wave through follicular atresia using a combination of estradiol benzoate and progesterone (P4) (Baruselli et al., 2017) or by inducing ovulation of a dominant follicle through the administration of GnRH (Wiltbank et al., 2015). Subsequently, it is necessary to control P4 concentrations so that, at the end of the protocol, P4 concentrations are minimal at the moment of AI. Thus, at the adequate moment, the intravaginal P4 device (exogenous source) is removed, and PGF2α is administered to induce luteolysis (endogenous source). Finally, ovulation synchronization is performed using estradiol esters or GnRH, allowing insemination to occur at a predetermined time (Baruselli et al., 2017). Additionally, equine chorionic gonadotropin (eCG) is included in the TAI protocol at the time of P4 device removal, with the aim of stimulating follicular growth, particularly in anestrous cows with low body condition score (BCS) (Sales et al., 2011) or in primiparous cows (Sales et al., 2016).

**Figure 1:**
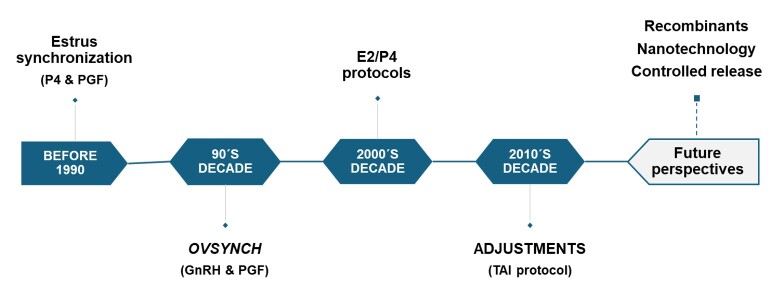
Timeline of the evolution of TAI protocols throughout the decades.

The main advantage of implementing hormonal treatments for TAI in cattle is to hasten the first ovulation in cows in postpartum anestrus or prepubertal heifers, consequently increasing reproductive efficiency (Bó et al., 2003). Furthermore, the adoption of TAI in livestock systems rationalizes reproductive management, enhances genetic gain, and improves reproductive efficiency. After establishing all these benefits and consolidating the technique, TAI protocols in beef and dairy cattle have resulted in pregnancies per AI (P/AI) ranging from 30 to 65% (Wiltbank et al., 2015; Sales et al., 2016; Baruselli et al., 2017). The optimization of TAI protocols to achieve satisfactory results was only possible due to numerous studies that have been and are being conducted to fine-tune the response at the protocol across different breeds, animal categories, and production systems. In this context, the objective of this review is to describe the evolution of studies related to TAI protocols throughout decades (1980-2020) in bovine females (Figure 1).

## Before 1990 - Estrus synchronization using progesterone and prostaglandin

In the beginning, studies aimed to synchronize or induce estrus in females with the intention of improving reproductive rates by increasing the service rate. In the 1940s and 1950s, the suppression of the estrous cycle was demonstrated in ewes (Dutt and Casida, 1948) and cows (Ulberg et al., 1951) after serial oral administrations of a progestin (Melengestrol Acetate - MGA) for long periods (14 to 18 days). Despite synchronizing estrus 3 to 7 days after the end of treatment, this strategy was not very effective in increasing fertility (Trimberger and Hansel, 1955) and it was observed that higher doses of progesterone, above 1 mg/day, resulted in longer intervals between the end of treatment and estrus (Christian and Casida, 1948; Willett, 1950; Ulberg et al., 1951). Despite the low fertility rates of estrus after P4 treatment, satisfactory conception rates were observed in the subsequent estrus (Zimbelman et al., 1970), demonstrating that hormonal supplementation of MGA alone was not able to increase fertility.

In the 1970s, the association between ingestion of MGA and administration of prostaglandin F2α (PGF2α) for estrus synchronization was reported. In cyclic cows, the supplementation with MGA for 9 days and administration of PGF2α on the last day of supplementation (D9) resulted in 94% estrus synchronization. Additionally, with this same treatment, a 66% response in estrus synchronization was achieved in cows in anestrus (Beal and Good, 1986). In comparison, the use of a progestogen in the form of an ear device (norgestomet) for 7 days followed by the simultaneous administration of PGF2α resulted in 76% of heifers in estrus 60 hours after its removal (Table 1; Heersche et al., 1979). In addition to PGF2α, the combination of P4 and estradiol (E2) resulted in estrus synchronization in heifers (Wiltbank and Zimmerman, 1962). Furthermore, the use of a norgestomet implant along with the administration of estradiol valerate was able to synchronize estrus in both non-cyclic and cyclic heifers (Wiltbank and Gonzalez-Padilla, 1975). At that same time, the administration of human chorionic gonadotropin (hCG) 48 hours after the removal of the MGA implant resulted in 100% ovulation after 40 hours in heifers. However, low conception rates (23%) were observed when AI was performed at predetermined times (4 and 24 hours after hCG) (Roche and Crowley, 1973). From these results, the search for hormonal associations to synchronize estrus and improve fertility rates began.

**Table 1:** Time to estrus (days) after progesterone and prostaglandin association treatments.

**Categories**	**Estrus time after P4 treatment**	**Reference**
Heifers	5 - 6 days	Christian and Casida (1948)
Cows	4.6 days - P/AI 12.5%	Trimberger and Hansel (1955)
Heifers	4 - 7 days	Willett (1950)
Heifers	5.2 days	Ulberg et al. (1951)
Heifers	60 hours - P/AI 62.2%	Heersche et al. (1979)

These studies suggested that the reduced fertility of heifers with synchronized estrus was related to the incidence of premature luteolysis and short cycles after treatments. Moreover, these unsatisfactory results may be related to ovulation of a persistent follicle and low oocyte quality (Yelich et al., 1997). In this context, other hormonal associations have been developed aiming to synchronize ovulation to perform AI at a predetermined time.

## The 90’s decade - The origin of the synchronization of ovulation protocol - *Ovsynch*

Despite the satisfactory results of previous studies in synchronizing estrus, the observation of estrus in cattle is a serious management problem. Studies have shown that the estrus observation rate was approximately 50% (Stevenson and Britt, 1977). This condition decreases reproductive efficiency in beef and dairy cows, increasing the period between calving and conception and, consequently, the interval between calvings. Some factors are directly related to the low estrus observation rate in cattle, such as the stage of the estrous cycle, milk yield, postpartum anestrus, duration and timing of estrus observation, ambient temperature, type of facility, management practices, the time an animal shows estrus, and the occurrence of nocturnal estrus (Galina et al., 1996; Pinheiro et al., 1998; Lopez et al., 2004). Thus, starting from the 1990s, studies began to focus on hormonal associations that would enable insemination at a predetermined time. Since, conventional artificial insemination after estrus observation is rarely used on beef and dairy farms due to prolonged postpartum anestrus in *Bos indicus* beef cows and low service rate in high-producing dairy cows (Baruselli et al., 2004).

Initially, in a robust study with dairy cows, AI was scheduled and performed after treatments with two doses of PGF2α (Archbald et al., 1992). However, the fertility results were not satisfactory due to the variability in the time to ovulation after PGF2α administration. After these studies, the first ovulation synchronization protocol with satisfactory results in dairy cows was Ovsynch. This protocol was developed after some studies that used GnRH to induce emergence of a new follicular wave (Macmillan and Thatcher, 1991; Wolfenson et al., 1994; Schmitt et al., 1996) with the administration of PGF2α, 7 days after GnRH (Thatcher et al., 1989; Twagiramungu et al., 1992). Thus, a protocol was proposed for dairy cows that consisted of administering an initial dose of GnRH (D0) and a dose of PGF2α seven days (D7) later. Next, a second administration of GnRH was performed 48 hours after PGF2α, and the TAI should be done 24 hours after the second dose of GnRH (Pursley et al., 1995). However, the ovulatory response to the first GnRH dose in this protocol is crucial for obtaining satisfactory conception rates and depends on the phase of the estrous cycle in which the female is. In this way, some studies have observed that the ideal time to start the Ovsynch protocol would be between days 5 and 8 of the estrous cycle (Vasconcelos et al., 1999; Moreira et al., 2000). This finding was responsible for the development of new research related to pre-synchronization protocols, aiming to increase the proportion of cows with ovulatory capacity at the beginning of the Ovsynch protocol.

The first strategy to increase the ovulatory response to the first GnRH in the Ovsynch protocol was the use of PGF2α, called Presynch-Ovsynch (Moreira et al., 2001). This strategy was based on the administration of two doses of PGF2α with a 14-day interval between them, and the start of Ovsynch 10 to 14 days after the second dose of PGF2α (Navanukraw et al., 2004). However, the result of this pre-synchronization was only satisfactory in cyclic cows, in addition to not synchronizing the ideal time to start the protocol (6 and 8 days of the estrous cycle), since the time between luteolysis (after PGF2α) and ovulation is variable according to the follicular diameter at the time of PGF2α treatment (Giordano et al., 2016). After these findings, a pre-synchronization using GnRH was developed and named G6G to pre-synchronize cows in anestrus. In this protocol, the females received an administration of PGF2α (D-8), 2 days later a dose of GnRH was administered (D-6), and 6 days after that, Ovsynch was initiated (D0) (Bello et al., 2006). Subsequently, other pre-synchronization methods were developed, with Double-Ovsynch being the most efficient protocol in inducing an ovulatory response to the first GnRH (Souza et al., 2008). The physiological concept of this pre-synchronization method is based on the strategy of inducing the formation of a pre-ovulatory dominant follicle that responds to the first GnRH of the protocol and inducing cyclicity in cows in anestrus before the Ovsynch protocol. Studies have shown that cows with corpus luteum (CL) at the beginning of the Ovsynch protocol have a greater conception rate (Herlihy et al., 2012). In this way, on Double-Ovsynch GnRH is administered on D0 and PGF2α 7 days later (D7). Next, a second dose of GnRH is administered 3 days later. Seven days after these treatments, a new Ovsynch protocol (GnRH + PGF2α + GnRH) is performed with the addition of TAI 16 hours after the second dose of GnRH. The Double-Ovsynch resulted in greater fertility rates compared to Presynch (Souza et al., 2008), both in primiparous and multiparous cows (Herlihy et al., 2012).

The use of P4 has also been reported in pre-synchronization protocols to improve the response to Ovsynch. Some studies used an intravaginal P4 device (CIDR) for 7 days between the two administrations of PGF2α (Presynch) (Chebel et al., 2006; Bicalho et al., 2007). The objective of inserting an exogenous source of P4 during presynchronization is also related to the increase in cows returning to cyclicity at the beginning of Ovsynch. However, no difference was observed in the conception rate of cows receiving or not the intravaginal P4 device (Bicalho et al., 2007). A few years later, our research group evaluated the use of P4 (P4synch Protocol) as a pre-synchronization method to the Ovsynch protocol (Silva et al., 2018), which consisted of inserting a P4 intravaginal device 10 days before the start of the Ovsynch protocol (D-10). The objective of this treatment was to induce the formation of a persistent dominant follicle responsive to GnRH at the beginning of the TAI protocol and to provide an environment with higher circulating P4 concentration during the initial development of the future ovulatory follicle, as the intravaginal device remained until the PGF2α administration (D7). The conception rate of the cows in the P4synch group was similar to that of the Double-Ovsynch protocol. However, it is important to highlight that the cost and time of implementing the P4synch protocol (21 days) is less than that of the Double-Ovsynch protocol (28 days). Recently, another pre-synchronization strategy with a P4 device was studied (Consentini et al., 2021). In this study, a P4 intravaginal device was inserted 15 days before the start of the Ovsynch protocol (D-15) and remained for 7 days. After this period (D-8), the device was removed and the cows received estradiol cypionate (EC) and PGF2α, with the aim of inducing the formation of a GnRH-responsive follicle from the beginning of the protocol, as well as increasing P4 concentrations during the protocol. On D0, the ovulation synchronization protocol was initiated, with the administration of GnRH along with the insertion of the P4 device. In addition to this modification to Ovsynch, an extra dose of PGF2α was implemented (D6 and D7). All the strategies and adjustments mentioned above were essential for the improvement of ovulation synchronization protocols that begin with GnRH (Table 2; Consentini et al., 2021). In recent decades, new strategies have been developed and will be discussed.

**Table 2:** Pregnancy per artificial insemination (P/AI) in dairy cows submitted to pre-synchronization protocols distinct from *Ovsynch*.

**Reference**	**n**	**P/AI, %**		***P***
Moreira et al. (2001)	185	36.0 (*Ovsynch*)	36.9 (*Presynch)*	> 0.05
Bello et al. (2006)	66	27.0 (*Ovsynch*)	50.0 (G6G)	0.08
Souza et al. (2008)	337	41.7 (*Presynch*)	49.7 (*Double-Ovsynch*)	0.03
Herlihy et al. (2012) ^*^	778	42.3 (*Presynch*)	52.5 (*Double-Ovsynch*)	0.02
Herlihy et al. (2012) +	909	34.3 (*Presynch*)	40.3 (*Double-Ovsynch*)	0.07
Bicalho et al. (2007)	1318	36.4 (*Presynch*)	34.5 (*Presynch* + P4)	> 0.05
Silva et al. (2018)	440	39.0 (*Double-Ovsynch*)	40.1 (P4synch)	0.85
Consentini et al. (2021)	909	-	42.7 (*Presynch* P4+EC)	
Consentini et al. (2025)	800	46.0 (*Double-Ovsynch*)	51.7 (*Pre* P4/E2)	0.22

* Primiparous; ^+^Multiparous.

Regarding beef cows, the fertility results of the Ovsynch protocol were unsatisfactory, especially in animals that are in anestrus (Barros et al., 2000). After these results, in the following years, research was directed towards the use of E2 esters in conjunction with P4 to synchronize the emergence of a new follicle.

## The 2000’S decade - E4/P4 protocols

### Association of progesterone and estradiol - emergence of new follicular wave

In Brazil, the 2000s were revolutionary for the implementation and use of TAI (Baruselli et al., 2025b). This fact was made possible by the development of protocols based on the administration of an estradiol ester simultaneously with the insertion of the P4 device. As mentioned earlier, GnRH is used at the beginning of the protocol to induce ovulation and, consequently, the emergence of a new follicular wave. With the same goal of synchronizing the onset of the follicular wave, studies have shown that the administration of E2 in association with P4 induces follicular atresia regardless of the follicle diameter (Silva et al., 2025) and, consequently, synchronizes the emergence of the follicular wave (Bó et al., 2003). Thus, the emergence of a new follicular wave occurs after the metabolism of estradiol and its suppressive effect on GnRH release. Thus, the follicular wave begins 2-5 days after the administration of this E2 ester (O’Rourke et al., 2000; Bó et al., 2002; Madureira et al., 2020).

The first study that associated P4 and E2 with positive fertility outcomes was known as “Syncro-Mate-B (SMB).” This protocol consisted of the insertion of a norgestomet ear implant for 9 days, simultaneously with the administration of estradiol valerate (EV) (Spitzer et al., 1978). Initially, the use of EV at the beginning of this protocol was to induce luteolysis in heifers that presented CL, thus resulting in a better response to estrus synchronization in these animals. Sometime later, it was discovered that the use of EV together with SMB could also induce the suppression of gonadotropin release, resulting in the emergence of a new follicular wave between 3 and 5 days after its administration (Bo et al., 1991). After these findings, studies began to use other E2 esters, in conjunction with P4, to better synchronize the emergence of the follicular wave and thus achieve synchronization of ovulation in female cattle (Baruselli et al., 2018b). The treatment with 17β estradiol was also tested at the beginning of the protocol. The hypothesis in the first experiment conducted was that 17β estradiol would more effectively induce the atresia of a dominant follicle when administered in association with norgestomet. Thus, after the administration of 17β estradiol alone, the LH peak and the drastic increase in FSH concentrations were observed, 12 hours after its decline. In contrast, when the ear device was inserted, there was no LH peak, and the release of FSH gradually increased between 24 and 42 hours after the metabolization of 17β estradiol, which occurs 6 hours after its administration. These findings demonstrated that the association between P4 and an E2 ester induces the suppression of gonadotropins, and that this suppression must last for 24 hours for the dominant follicle to undergo atresia (Bo et al., 1994).

The use of estradiol benzoate (EB; 2 mg) at the beginning of the protocol along with the exogenous source of P4 also had satisfactory results in synchronizing the emergence of the follicular wave 4 days after its administration (Bridges et al., 1999). In the initial studies, gelatin capsules containing EB were used, inserted together with the intravaginal P4 device (Macmillan and Peterson, 1993; Macmillan and Burke, 1996). However, it was observed that the intramuscular (IM) administration of EB resulted in better synchronization of the follicular wave (Bó et al., 1996). Thus, currently, the use of IM injection of EB at the beginning of the protocol is consolidated and presents satisfactory results (Sales et al., 2024a). However, recently, our research group resumed studies using EV together with P4 at the beginning of the ovulation synchronization protocol (Sales et al., 2024b). In this study, similar fertility results to protocols initiated with EB were observed when EV was combined on D0 and EC on D9, to synchronize ovulation and the TAI to be performed 48 hours after the device was removed (D0: EV + P4; D9: EC + eCG; D11: TAI). Despite similar results in fertility, E2 esters have different pharmacokinetics and half-life (EV: 7-8 days; EB: 3 days; EC: 10-12 days) (Colazo et al., 2003; Sales et al., 2024b).

### Controlling circulating P4 concentrations

The consolidation of the use of intravaginal P4 devices in the ovulation synchronization protocol also occurred in the 2000s. During the protocol, it is necessary to control the concentrations of P4 for a certain period to avoid premature ovulations, control the final growth of the dominant follicle, and, after its abrupt drop, allow ovulation. In this context, one of the advantages of using intravaginal devices with an appropriate concentration is the controlled release of P4 (Bó et al., 2003). To obtain satisfactory results, the devices must remain in the bovine females for 5 to 9 days, and after this period, it is necessary to reduce the P4 concentrations to allow true proestrus, characterized by high E2 concentrations and low P4 concentrations. However, the duration of P4 exposure (protocol duration; D7, D8, or D9) depends on the animal category. In primiparous cows or in cows with low body condition (≤ 2.5 on a scale of 1 to 5), it is necessary to maintain the P4 device for a longer period to allow for greater follicular growth and greater rates of ovulation and conception, due to the lower rate of follicular growth (Carvalho et al., 2023b). However, in multiparous cows, despite the lower expression of estrus in the 7-day protocol, the conception rate was similar among the different days of protocol duration (D7, D8, and D9). However, in the 7-day protocols, it is important to administer GnRH at the time of TAI (Prata et al., 2020). Another exogenous source of P4 was tested by our research group at the beginning of the protocol in association with EB. In this study, the use of 75 mg of long-acting injectable progesterone (P4i) was not a viable alternative to replace the intravaginal P4 device, as the conception results were inferior (Carvalho et al., 2023a). Thus, in protocols that combine progesterone and estradiol, the use of the P4 device is still indispensable for promoting ovulation synchronization.

In Ovsynch-type protocols, it is also possible to include an exogenous source of P4. Supplementation with P4 in this type of protocol improves the synchronization of wave emergence and oocyte quality (Consentini et al., 2021). In Brazil, due to the approval of the use of E2 esters, the protocol commonly used in dairy cows begins with the administration of GnRH, EB, and the insertion of the P4 device. Studies have shown that protocols initiated with GnRH or GnRH/EB and P4 presented better conception rates in lactating dairy cows compared to the use of EB alone (Pereira et al., 2015; Carneiro et al., 2017; Tschopp et al., 2022, 2024). However, after the administration of GnRH on D0, it is necessary to administer 2 doses of PGF2α to achieve complete luteolysis and minimal P4 concentrations at the time of AI (Borchardt et al., 2018). This addition of a second dose of PGF2α is necessary because, after the administration of GnRH on D0, ovulation and the formation of CL are expected to occur, which, in the initial phase, may not respond to just one treatment with PGF2α (Nascimento et al., 2014).

### Equine chorionic gonadotropin (eCG)

The TAI protocols have developed significantly, allowing cows to be inseminated without the need for estrus observation. However, the conception results varied greatly depending on the body condition and farm management. This inconsistency in results at the beginning of the use of TAI prevented this biotechnology from spreading among a greater number of farms in Brazil. As research advanced, it was observed that the main problem in *Bos indicus* beef cows was the low LH pulsatility in the early postpartum period, mainly in low body condition and primiparous cows. Due to this condition, an alternative was sought to improve the final growth of the dominant follicle under low LH pulsatility conditions. In this context, equine chorionic gonadotropin (eCG) produced in the endometrial cups of pregnant mares emerges as a promising molecule. The use of eCG at the time of removal of the P4 device improved the fertility in early postpartum anestrus cows, with a low BCS (< 2.75 on a scale of 1 to 5) and also in cows with compromised growth of the dominant follicle (Sales et al., 2011). The benefits of eCG in these females are related to the pattern of gonadotropin release in the postpartum period, especially in *Bos indicus* cows. In these animals, the presence of the calf after birth and malnutrition affect LH pulsatility and, consequently, the final growth of the dominant follicle and ovulation (Jolly et al., 1995; Yavas and Walton, 2000). Initially, to circumvent the effect of the calf's presence, a temporary separation (48 hours) between the cow and the calf was carried out to reduce the negative feedback induced by the release of endogenous opioids that block the release of GnRH and, consequently, LH (Williams et al., 1983; Edwards, 1985). However, due to the management difficulties in implementing this separation, the use of eCG became an excellent alternative to stimulate the final growth of the dominant follicle because of its FSH/LH-like action, serving as an important hormonal support for the final development of the follicle (Murphy and Martinuk, 1991). Studies have shown that eCG treatment increases daily (0.55 mm) follicular growth before ovulation and, consequently, the rates of ovulation and pregnancy (Control - growth rate of 0.90 mm/day and eCG growth rate of 1.45 mm/day) (Sales et al., 2011). Overall, the effects of eCG on follicular dynamics and fertility were observed in different categories of animals. However, in primiparous cows, its effect is more pronounced (Sales et al., 2016).

The commonly eCG dose used in *Bos indicus* cows is 300 IU (Simões et al., 2018; Prata et al., 2020; Alves et al., 2021). However, in primiparous cows, an extra eCG administration was proposed (one dose 2 days before and another at the time of P4 device removal). This strategy resulted in a 6.8% increase in P/AI compared to the administration of a single dose at the time of P4 device removal (Pugliesi et al., 2022). In agreement with these results, another study demonstrated that splitting the dose or increasing it to 400IU improved fertility outcomes in primiparous cows with BCS ≤ 2.75, but no effect was detected in multiparous cows (Sales et al., 2024c). However, other studies have shown that increasing the dose to 400 IU did not improve follicular growth or the conception rate of *Bos indicus* cows with low body condition (BCS < 2.5, scale of 1 to 5) (Sales et al., unpublished data). In *Bos indicus* heifers, a similar fertility outcome was observed in protocols that use eCG (higher ovulation rate to the protocol, greater P/AI, and bigger CL after TAI). However, no differences were observed in these parameters when using the dose of 200 IU compared to 300 IU (Felisbino et al., 2024), suggesting that, in heifers, a lower dose of eCG (200 IU) can be used. The results on the use of eCG in lactating dairy cows are contradictory, with improvements in pregnancy rates in pasture managed dairy cows (Bryan et al., 2010, 2013) and limited or no improvements in high-producing intensively managed herds.

### Ovulation inducers

The last premise of the TAI protocol is to synchronize ovulation. As mentioned earlier, in the first studies related to hormonal administrations the goal was to synchronize estrus and perform the AI after observing estrus. After the hormonal adjustments were made, it was possible to synchronize ovulation and inseminate the female cattle at a predetermined time. The synchronization of ovulation can be performed using E2 esters (Melo et al., 2016) or GnRH (Pursley et al., 1995). However, despite the use of GnRH inducing a better synchronized ovulation than E2 esters (Pancarci et al., 2002; Souza et al., 2009), a lower incidence of expression of estrus is observed (Sá et al., 2011; Consentini et al., 2021). In *Bos indicus* cows, the lower expression of estrus was negatively correlated with fertility (Sá et al., 2011) and positively associated with pregnancy loss (Consentini et al., 2023). Furthermore, to achieve satisfactory results in Ovsynch-type protocols, GnRH administration must be performed 16 hours before AI, requiring additional management. In this context, E2 esters (EB or EC) are widely used options in Brazil as ovulation inducers in beef and dairy cattle reproduction. However, there are pharmacokinetic differences between EB and EC that result in different intervals to the LH peak after their administration. The LH peak in cows that received EB occurred 31 hours earlier than in cows treated with EC (Sales et al., 2012). Due to this pharmacological characteristic, EC administration can be performed at the time of P4 device removal, while EB should be administered 1 day later so that ovulations occur, on average, 70 hours after P4 device removal. In this study, the fertility results were similar between the two E2 esters. However, the use of EB 1 day after the removal of the P4 device requires additional management, as well as the use of GnRH (Sales et al., 2012). The effect of the association of EC at the time of P4 removal and GnRH 16 hours before AI in the fertility of dairy cows was evaluated. This strategy showed similar results when only EC or GnRH was used as ovulation inducers (Consentini et al., 2021). In this context, the use of EC as an ovulation inducer is recommended because it does not require additional management and presents satisfactory results in dairy and beef cows.

## The 2010’S decade - Adjustments to the synchronization protocol

### Resynchronization protocols

The increase in productivity on beef farms that adopt TAI is related to the anticipation of the first ovulation after calving, the concentration of calving and weaning of calves at more appropriate times. In these systems, a 12-month calving interval is sought to increase the efficiency of beef and dairy cattle farming. However, the prolonged anestrus period after calving in these females makes it impossible to achieve this goal, as the cows would need to conceive within 72 days after calving to obtain one calf per year (gestation period of 293 days in *Bos indicus*) (Monteiro et al., 2023).The positive effects of ovulation synchronization strategies were reported in a study that compared reproductive programs using only natural mating (NM) or TAI followed by NM (TAI + NM) during a 90-day breeding season in *Bos indicus* cows (Sá et al., 2013). In this experimental model, a greater (P = 0.001) pregnancy rate at 45 days was observed when these females were subjected to TAI+NM (63.5%) compared to TAI only. However, at the end of the breeding season, the proportion of pregnant females was similar between the reproductive programs (TAI+NM: 77.0% vs. NM: 71.0%; P = 0.31). In this last analysis, it should be taken into consideration that cows subjected to TAI+NM had fewer days until conception (11 days from TAI) compared to cows exposed to NM (55 days). This observation is important because cows that calve at the beginning of the calving season wean heavier calves and exhibit greater reproductive efficiency in the subsequent breeding season (Funston et al., 2012). In this context, resynchronization protocols have been developed and have been widely used to further intensify herd production (Pugliesi et al., 2024). Currently, the interval between the first AI and the pregnancy diagnosis (PD) is variable and PD can be performed by ultrasound in B-mode (30 days after AI) or in Doppler mode (20 to 22 days after AI). In conventional resynchronization strategy, the PD is performed 30 days after TAI and non-pregnant cows are subjected to a new ovulation synchronization protocol (Marques et al., 2015). In early resynchronization, the P4 device is inserted and EB (2mg) is administered to all cows (pregnant and non-pregnant) 22 days after the TAI, and at the time of P4 device removal (30 days after the TAI), PD is performed (Sá et al., 2014). Finally, in super-early resynchronization, the females are subjected to the TAI protocol before the PD (14 days after the TAI), however, the PD is performed by visualizing the blood perfusion of the CL in Color Doppler mode ultrasound (22 days after TAI) (Sá et al., 2014; Pugliesi et al., 2024). Thus, it is possible to perform three AI sessions in 48, 64 and 84 days using super-early, early and traditional resynchronization, respectively (Baruselli et al., 2018a). In addition to the anticipation of conception, the implementation of resynchronization programs increases reproductive efficiency due to the large number (63%) of *Bos indicus* cows that remain in anestrus after the first AI. A 10 percentage points increase in pregnancy rate at the end of the breeding season was observed when more than one ovulation synchronization protocol was performed (1X AI - 77.1%; 2X AI - 86.3% and 3X AI - 87.4%) (Crepaldi et al., 2014). In addition to this increase in fertility, when three TAI protocols are performed, it may eliminate the need to use NM. In this way, these strategies allow for accelerating the genetic gain of the herds, concentrating the calving season, and producing heavier calves at weaning.

### Long-acting injectable progesterone prior to the ovulation synchronization protocol

Prolonged postpartum anestrus negatively affects the reproductive indices of cattle herds (Montiel and Ahuja, 2005), especially in *Bos indicus* cows, where this mechanism is accentuated by the presence of the calf (Williams et al., 1983) and inadequate nutrition (Diskin et al., 2003). The pulsatility of LH in females under these conditions is compromised, resulting in follicular growth without subsequent ovulation (Crowe et al., 2014). Some strategies have been tested with the aim of stimulating LH release in the early postpartum period, such as the use of P4. In anestrus cows, P4 acts on the hypothalamus by reducing the expression of E2 receptors, decreasing the negative feedback on the production and release of GnRH and, consequently, LH (Day and Anderson, 1998). During ovulation synchronization protocols, supplementation with P4 (intravaginal device) sometimes is insufficient to stimulate adequate LH release, especially in cows with low BCS and heifers. In these animals, the final growth of the dominant follicle is impaired, resulting in small follicles with a low ovulation rate. In ovulation synchronization protocols, it has been reported that 21% of the cows do not adequately respond with the presence of an ovulatory follicle before AI (Sales et al., 2016). Due to the number of animals that do not respond to the hormonal stimulation of the protocol, strategies (nutritional or supplementation with P4) that could improve these results were studied.

In recent years, our research group has evaluated the treatment with P4i prior to the TAI protocol on the fertility of bovine females after calving. In these studies, it was observed that *Bos indicus* beef cows that received 150 mg of P4i had 1.68 times more chance of conceiving after TAI (Simões et al., 2018). Similar results were observed in *Bos taurus* beef cows, where supplementation with 150 mg of P4i prior to the AI protocol (D-10) increased the conception rate by 9 percentage points (Simões et al., 2024), and Holstein/Jersey dairy cows, where the administration of 300 mg of P4i (D-7) increase conception rate by 7 percentage points (Simões et al., 2022). This result was more pronounced in cows that did not have a CL at the time of P4i administration (Control = 43.0% vs P4i = 58.0%, P = 0.001). Furthermore, P4i was also previously used in resynchronization protocols (22 days after TAI). However, no difference was observed between the experimental groups (Gonçales et al., 2024). Under the conditions of this study, the cows were already exposed to an exogenous P4 source during the first TAI, which possibly stimulated the hypothalamic system to return to cyclicity. Finally, it has been reported recently that the administration of P4i 10 days prior to the initiation of a synchronization protocol for timed-embryo transfer (TET) increased the fertility of embryo recipient cows (Rodrigues et al., 2024). Thus, supplementation with P4 prior to the ovulation synchronization protocol for AI or ET is an interesting strategy to increase the reproductive efficiency of the biotechnology used.

### Protocols without E2 on *Bos indicus* cows

The ovulation synchronization protocols that associate E2 and P4 are well established, and their use is recommended for *Bos indicus* cows in Brazil. However, in some countries, the use of E2 esters in ovulation synchronization protocols is prohibited, and recently, there has been a restriction on the export of meat from Brazil to the European Union from herds that use this hormone in female cattle. As mentioned earlier, Ovsynch-type protocols show satisfactory fertility results in dairy cows. In contrast, in *Bos indicus* cows, fertility results are unsatisfactory, especially in animals that are in anestrus (Barros et al., 2000). In this context, some research groups are developing strategies to improve the non-estrogen-based TAI protocol for *Bos indicus* beef cows, in case its prohibition affects national productivity. A recent study by our research group compared the fertility of *Bos indicus* cows subjected to the P4/E2-based protocol with a protocol without E2. The females that received GnRH (buserelin) on D0 exhibited a larger follicular diameter on D8 and a greater ovulation rate on D0 compared to the animals that received EB. However, the cows that received EC at the time of the removal of the P4 device had a greater incidence of expression of estrus. In addition, the conception rate was greater in the control group cows. Thus, it was observed that the removal of E2 (EC and EB) in ovulation synchronization protocols reduces the fertility of lactating *Bos indicus* cows (Lemos et al., unpublished data). After these results, a new hypothesis will be tested by our research group, using a more potent GnRH analog (Deslorelin).

### Protocols without intravaginal P4 device

Currently, the most used exogenous source of P4 in TAI protocols are the intravaginal devices that contain different concentrations of P4 and result in an immediate increase in the circulating concentration of P4 (Sales et al., 2015, 2021). However, there are concerns related to animal welfare and the environment, due to possible discomfort/afflictions in the reproductive tract of female cattle and the disposal of the material with P4, respectively. During the TAI protocol, it is necessary to control the concentrations of P4 for a determined period. This control can be carried out by an exogenous or endogenous source of P4. The P4i, described earlier, increased the fertility of female cattle when administered prior to the TAI protocol. However, when this exogenous source of P4 was used as a substitute for the intravaginal device, there was a reduction in the conception rate of lactating *Bos indicus* cows (Control = 71.7 vs P4i = 27.0; P = 0.0001) (Carvalho et al., 2023a). The differences between the exogenous sources of P4 (intravaginal device vs. injectable) may be related to variations in dry matter intake and hepatic metabolism of this steroid (Sangsritavong et al., 2002). Thus, the concentration of P4 after its administration in injectable form depends on the condition of the bovine female (lactating vs. non-lactating), a fact that is not as relevant when using intravaginal devices. In this context, our research group is seeking a strategy to induce the formation of a CL at the beginning of the TAI protocol, so that the P4 concentration necessary to control follicular growth comes from an endogenous source, as in the conventional Ovsynch-type protocol.

## Future perspectives

Despite the positive results observed in hormonal associations over the years to improve the reproductive efficiency of female cattle, some adjustments are still necessary. Currently, recombinant hormones produced by genetic engineering have emerged as a substitute for the large-scale production of products previously derived from animals (Baruselli et al., 2023). Among them, eCG stands out, which is originally extracted from the endometrial cups of pregnant mares and plays a very important role in TAI protocols in *Bos indicus* cows. In a recent study, similar fertility results were observed in *Bos indicus* cows subjected to a TAI protocol using eCG or its recombinant molecule (Villarraza et al., 2021; Abreu et al., 2023; Cattaneo et al., 2024). In addition to this glycoprotein, promising results related to reproductive efficiency were observed in cows treated with recombinant FSH and recombinant bovine somatotropin (Rebeis et al., 2023; Rodrigues et al., 2023). Adding to the production of recombinant hormones, the future development of new drugs with controlled release targeted at specific organs using nanotechnology is expected, which could increase the reproductive efficiency of ovulation synchronization protocols and reduce labor and cow stress, allowing for the optimization of cattle production systems.

## Conclusion

Over the years, studies have focused on increasing productivity, profitability, and sustainability of cattle farming, with an emphasis on reproductive efficiency. In this context, ovulation synchronization protocols were a milestone in bovine reproduction, as TAI protocols allowed genetic (qualitative) associated with economic (quantitative) gains through increased fertility in female cattle. In this way, with this biotechnology, it was possible to significantly increase the use of AI with greater production of milk and beef in smaller areas, preserving the environment. In the last 30 years, the TAI protocols have been improved, and the results have become more reliable and consistent. However, fine adjustments are still necessary, such as the use of P4i prior to the TAI protocol to further increase the fertility of *Bos indicus* and *Bos taurus* cows. Similarly, the use of recombinant drugs and nanotechnology could become viable strategies to optimize AI protocols and, consequently, the fertility of female cattle.

## Data Availability

Research data is available in the body of the article.
